# Heat-Stress Impacts on Developing Bovine Oocytes: Unraveling Epigenetic Changes, Oxidative Stress, and Developmental Resilience

**DOI:** 10.3390/ijms25094808

**Published:** 2024-04-28

**Authors:** Xiaoyi Feng, Chongyang Li, Hang Zhang, Peipei Zhang, Muhammad Shahzad, Weihua Du, Xueming Zhao

**Affiliations:** 1 Institute of Animal Sciences (IAS), Chinese Academy of Agricultural Sciences (CAAS), Beijing 100193, China; 17806257712@163.com (X.F.); 15652652378@163.com (C.L.); 82101215397@caas.cn (H.Z.); 82101209707@caas.cn (P.Z.); mshahzad@niab.org.pk (M.S.); duweihua@caas.cn (W.D.); 2 College of Animal Science and Technology, Qingdao Agricultural University (QAU), Qingdao 266000, China

**Keywords:** heat stress, bovine, oocytes, epigenetic changes, developmental resilience

## Abstract

Extreme temperature during summer may lead to heat stress in cattle and compromise their productivity. It also poses detrimental impacts on the developmental capacity of bovine budding oocytes, which halt their fertility. To mitigate the adverse effects of heat stress, it is necessary to investigate the mechanisms through which it affects the developmental capacity of oocytes. The primary goal of this study was to investigate the impact of heat stress on the epigenetic modifications in bovine oocytes and embryos, as well as on oocyte developmental capacity, reactive oxygen species, mitochondrial membrane potential, apoptosis, transzonal projections, and gene expression levels. Our results showed that heat stress significantly reduced the expression levels of the epigenetic modifications from histone H1, histone H2A, histone H2B, histone H4, DNA methylation, and DNA hydroxymethylation at all stages of the oocyte and embryo. Similarly, heat stress significantly reduced cleavage rate, blastocyst rate, oocyte mitochondrial-membrane potential level, adenosine-triphosphate (ATP) level, mitochondrial DNA copy number, and transzonal projection level. It was also found that heat stress affected mitochondrial distribution in oocytes and significantly increased reactive oxygen species, apoptosis levels and mitochondrial autophagy levels. Our findings suggest that heat stress significantly impacts the expression levels of genes related to oocyte developmental ability, the cytoskeleton, mitochondrial function, and epigenetic modification, lowering their competence during the summer season.

## 1. Introduction

Over the last few decades, there has been a noticeable increase in milk and milk byproducts on the market [1]. This societal inclination has emerged due to recognition of the dietary benefits of dairy products for human physical health and well-being [2]. Therefore, to meet the escalating market demand, it is imperative to upscale dairy cattle productivity [3]. Production of the cow is directly correlated to reproductive performance [4]. However, dairy cow reproduction is influenced by many external factors, including nutrition, management, and environmental conditions, and the environmental factor has a vivid impact [5]. Currently, the dairy industry is confronted with substantial difficulties arising from adverse environmental circumstances because of global climate change [6].

Dairy cow conception rates fall by 20% to 30% on average throughout the summer, whereas they drip significantly by almost 50% during the peak days [7]. Cattle can surpass the thermoneutral zone and be unable to dissipate excessive body heat to maintain their thermal balance in the summer due to extreme ambient temperatures [4]. This situation causes extreme heat stress to the animal [8]. Under severe heat stress, the dietary intake of cattle is reduced, resulting in decreased daily weight gain and milk production [9]. One of the most important effects of heat stress on animal husbandry is the reduction of reproduction rate in cattle, as well as a huge economic loss to the dairy industry [5]. With global warming temperatures rising every year, the detrimental impacts of heat stress on cattle fertility are becoming an increasingly urgent problem [10].

Various studies have reported the adverse impact of heat stress on the fertility of cattle [11], sheep [12], pigs [13], mice [14], and rabbits [15]. In cattle, the negative impact of heat stress on estrus, uterine function, follicular dynamics, and oocyte development and survival has been well-studied [5]. Among the aforementioned processes, oocyte development capacity is considered a key limiting factor that determines fertility [16]. Heat stress directly affects oocyte structure and development by affecting ovulation, impairing mitochondrial function, reducing mitochondrial DNA copy number, and decreasing mitochondrial ability to generate ATP and membrane potential [11,17]. Consequently, under vulnerable conditions, elevated stress negatively impacts high producer fertility [11]. 

Heat stress has been linked to epigenetic modifications that adversely affect the growth and development of bovine oocytes and embryos [18]. The dynamic changes in the epigenome are caused by the complex interaction of genetic and environmental factors [19]. They are considered very critical for normal dairy cattle oocyte development, particularly when exposed to heat stress [20]. Epigenetic modifications are molecular modifications that regulate gene expression without altering the DNA sequence and can be passed down to future generations [21,22]. These modifications mainly include DNA methylation, histone modifications, chromatin remodeling and non-coding RNA regulation [23]. Meanwhile, histone modifications and DNA methylation play a critical role in gene expression [24] and epigenetic reprogramming [25]. Histones are a group of proteins (H1, H2A, H2B, H3 and H4), and the core histone H2A, H2B, H3 and H4 octamers wrap around DNA to form nucleosomes, which are the basic units of chromatin [26]. DNA methylation takes place mainly at the fifth of the cytosine residues of the DNA sequence carbon with the addition of methyl or hydroxymethyl, 5-methylcytosine (5mC) and 5-hydroxymethylcytosine (5hmC), respectively [24]. DNA methylation is mediated by the DNA methyltransferase family [27] and is considered the most stable epigenetic modification in most mammalian genomes [20]. The cellular mechanisms underlying the impact of heat stress on oocyte developmental competence have been well explored, yet few studies have investigated the epigenetic modification mechanisms involved [11].

Environmental factors and epigenetics play a crucial role in determining the fertility of dairy cows. Therefore, this study aimed to investigate the impact of heat stress on epigenetic modifications in oocytes and embryos, as well as its effects on reactive oxygen species (ROS), mitochondrial membrane potential (ΔΨm), mitochondrial distribution, ATP levels, mitochondrial DNA copy number (mtDNA), mitochondrial autophagy, apoptosis, and transzonal projections (TZPs) in oocytes. In addition, the effects of heat stress on oocyte developmental competence, cytoskeleton, mitochondrial function, and expression levels of genes related to epigenetic modification were also explored to understand the underlying mechanism of action of heat stress. The study results will offer deep insights into factors that affect dairy cow fertility and aid in managing their reproductive health during extreme summer.

## 2. Results

### 2.1. Effects of Heat Stress on Oocyte and Embryo Development

Referring to the Table 1, the maturation rate of the heat-stress group (61.99 ± 5.46%) was significantly lower than that of the control group (84.07 ± 7.52%), the cleavage rate of the heat-stress group (58.91 ± 2.95%; *p* < 0.05) was significantly lower than that of the control group (80.32 ± 1.75%; *p* < 0.05), and the blastocyst rate (23.25 ± 1.56%) was also significantly lower than that of the control group (42.04 ± 1.06%; *p* < 0.05). 

### 2.2. Effect of Heat Stress on Histone Modification

#### 2.2.1. Effect of Heat Stress on the Expression Level of Histone H1 during Oocyte Development

**Figure 1:**
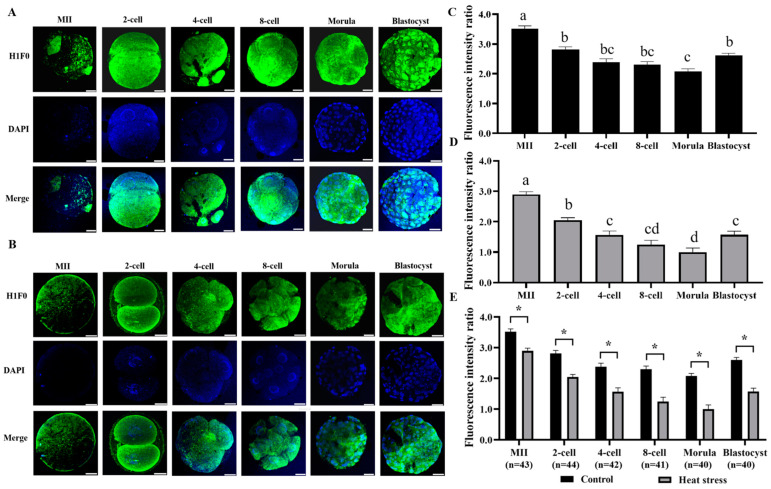
Effect of heat stress on fluorescence intensity of histone H1F0 in bovine oocytes and embryos. Secondary antibody Alexa Fluor-488 (anti-rabbit) binds to primary antibody H1F0 with green fluorescence. (**A**) Representative image staining of control group histone H1F0 at various cell stages of bovine oocyte development. Bar = 50 μm. (**B**) Representative image staining of heat-stress group histone H1F0 at various cell stages of bovine oocyte development. Bar = 50 μm. (**C**) Expression levels of each cell stage in the control group. (**D**) Expression levels of each cell stage in the heat-stress group. Values with no common superscript lowercase letters mean significant differences between groups (*p*  <  0.05). (**E**) Effect of heat stress on the expression level of histone H1F0 in bovine oocytes and embryos. The asterisk (*) denotes significant disparities between groups (*p* < 0.05).

The results of histone H1F0 staining in both control and heat-stressed oocytes and embryos are displayed in Figure 1A,B. The statistical analysis of the histone H1 expression levels in the control and heat-stressed oocytes and embryos is shown in Figure 1E. Comparing the heat-stressed to the control, the results showed a significantly lower histone H1 fluorescence intensity at all developmental stages (*p* < 0.05).

#### 2.2.2. Effect of Heat Stress on the Expression Level of Histone H2A during Oocyte Development

**Figure 2:**
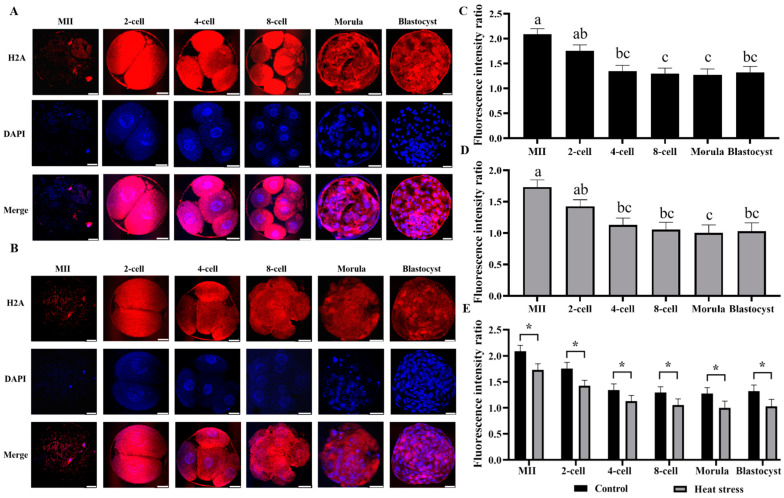
Effect of heat stress on fluorescence intensity of histone H2A in bovine oocytes and embryos. Secondary antibody Alexa Fluor-647 binds to primary antibody H2A with red fluorescence. (**A**) Representative image staining of control group histone H2A at various cell stages of bovine oocyte development. Bar = 50 μm. (**B**) Representative image staining of heat-stress group histone H2A at various cell stages of bovine oocyte development. Bar = 50 μm. (**C**) Expression levels of each cell stage in the control group. (**D**) Expression levels of each cell stage in the heat-stress group. Values with no common superscript lowercase letters mean significant differences between groups (*p*  <  0.05). (**E**) Effect of heat stress on the expression level of histone H2A in bovine oocytes and embryos. The asterisk (*) denotes significant disparities between groups (*p* < 0.05).

The results of histone H2A staining in both control and heat-stressed oocytes and embryos are depicted in Figure 2A,B. The statistical analysis of the histone H2A expression levels in both groups is shown in Figure 2E. The results showed that the heat-stressed group had significantly less histone H2A fluorescence intensity than the control during all stages of development (*p* < 0.05).

#### 2.2.3. Effect of Heat Stress on the Expression Level of Histone H2B during Oocyte Development

**Figure 3:**
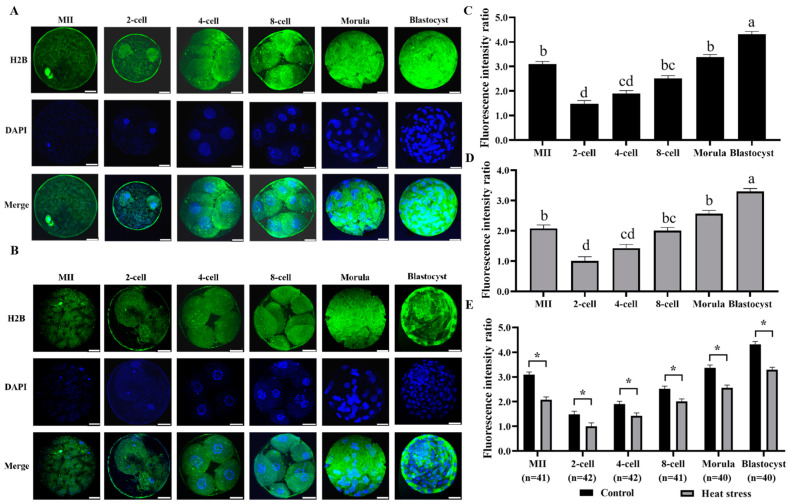
Effect of heat stress on fluorescence intensity of histone H2B in bovine oocytes and embryos. Secondary antibody Alexa Fluor-488 (anti-mouse) binds to primary antibody H2B with green fluorescence. (**A**) Representative image staining of control group histone H2B at various cell stages of bovine oocyte development. Bar = 50 μm. (**B**) Representative image staining of heat-stress group histone H2B at various cell stages of bovine oocyte development. Bar = 50 μm. (**C**) Expression levels of each cell stage in the control group. (**D**) Expression levels of each cell stage in the heat-stress group. Values with no common superscript lowercase letters mean significant differences between groups (*p*  <  0.05). (**E**) Effect of heat stress on the expression level of histone H2B in bovine oocytes and embryos. The asterisk (*) denotes significant disparities between groups (*p* < 0.05).

Figure 3A,B show the histone H2B staining of both control and stress-induced groups. The statistical analysis of the histone H2B expression levels in both groups is shown in Figure 3E. When comparing the heat-stressed group to the control, the results showed a significant decline in the histone H2B fluorescence intensity at all developmental stages (*p* < 0.05).

#### 2.2.4. Effect of Heat Stress on the Expression Level of Histone H4 during Oocyte Development

**Figure 4:**
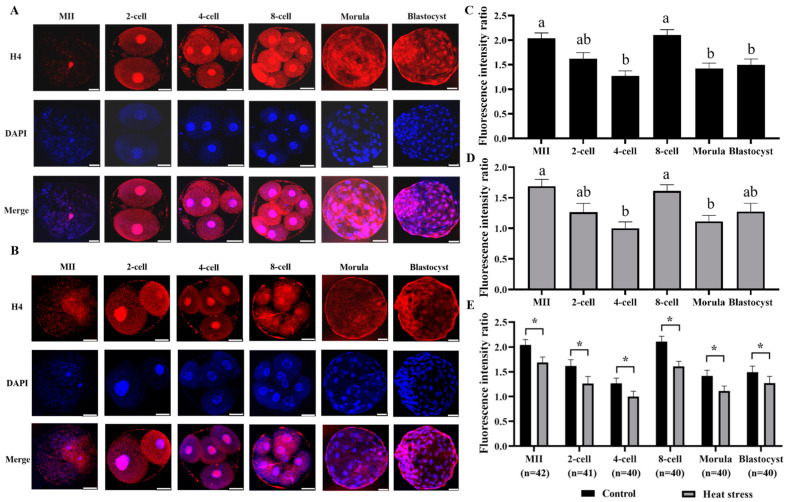
Effect of heat stress on fluorescence intensity of histone H4 in bovine oocytes and embryos. Secondary antibody Alexa Fluor-680 binds to primary antibody H4 with red fluorescence. (**A**) Representative image staining of control group histone H4 at various cell stages of bovine oocyte development. Bar = 50 μm. (**B**) Representative image staining of heat-stress group histone H4 at various cell stages of bovine oocyte development. Bar = 50 μm. (**C**) Expression levels of each cell stage in the control group. (**D**) Expression levels of each cell stage in the heat-stress group. Values with no common superscript lowercase letters mean significant differences between groups (*p*  <  0.05). (**E**) Effect of heat stress on the expression level of histone H4 in bovine oocytes and embryos. The asterisk (*) denotes significant disparities between groups (*p* < 0.05).

The results of histone H4 staining in both control and stressed groups are displayed in Figure 4A,B. The statistical analysis of the histone H4 expression levels in the control and heat-stress groups of oocytes and embryos is presented in Figure 4E. The results showed that the heat-stressed group had significantly lower histone H4 fluorescence intensity than the control at all stages (*p* < 0.05).

### 2.3. Effects of Heat Stress on DNA Methylation and DNA Hydroxymethylation

#### 2.3.1. Effect of Heat Stress on the Expression Level of DNA Methylation during Oocyte Development

**Figure 5:**
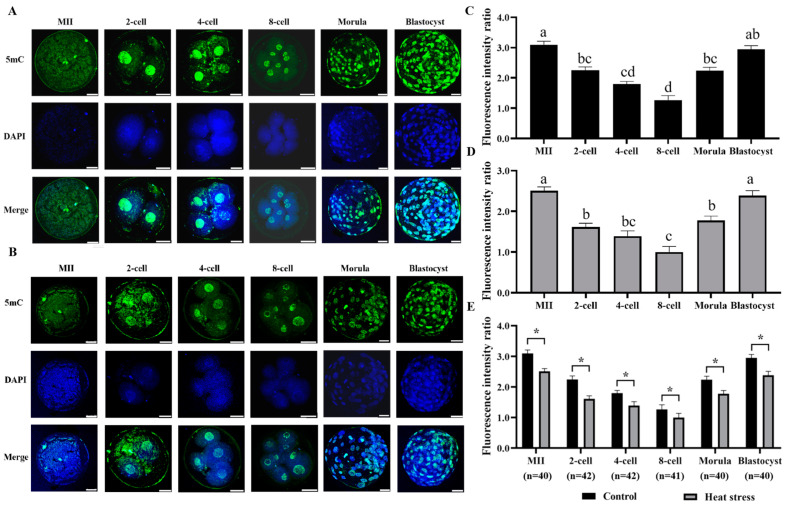
Effect of heat stress on fluorescence intensity of 5mC in bovine oocytes and embryos. Secondary antibody Alexa Fluor-488 (anti-mouse) binds to primary antibody 5-methylation with green fluorescence. (**A**) Representative image staining of control group 5mC throughout various phases of oocyte development. Bar = 50 μm. (**B**) Representative image staining of heat-stress group 5mC throughout different phases of oocyte development. Bar = 50 μm. (**C**) Expression levels of each cell stage in the control group. (**D**) Expression levels of each cell stage in the heat-stress group. Values with no common superscript lowercase letters mean significant differences between groups (*p*  <  0.05). (**E**) Effect of heat stress on the expression level of 5mC in bovine oocytes and embryos. The asterisk (*) denotes significant disparities between groups (*p* < 0.05).

DNA methylation (5mC) staining of control and heat-stressed oocytes and embryos is shown in Figure 5A,B. Figure 5E shows the statistical analysis of the results of DNA methylation expression levels in oocytes and embryos of the control and heat stress-induced groups. The outcomes reveal that compared to the control, fluorescence intensity at 5mC was significantly low during the heat stress at all cellular developmental stages (*p* < 0.05).

#### 2.3.2. Effect of Heat Stress on the Expression Level of DNA Hydroxymethylation during Oocyte Development

**Figure 6:**
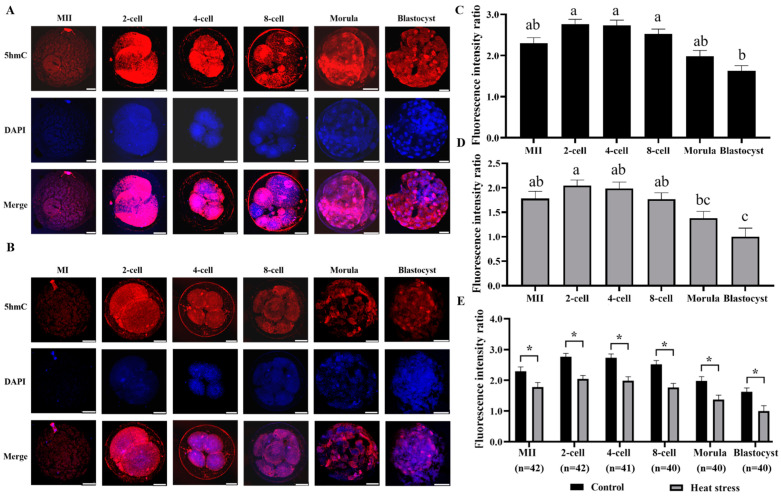
Effect of heat stress on fluorescence intensity of 5hmC in bovine oocytes and embryos. Secondary antibody Alexa Fluor-594 binds to primary antibody 5-hydroxymethylation with red fluorescence. (**A**) Representative image staining of control group 5hmC at various cell stages of bovine oocyte development. Bar = 50 μm. (**B**) Representative image staining of heat-stress group 5hmC at various cell stages of bovine oocyte development. Bar = 50 μm. (**C**) Expression levels of each cell stage in the control group. (**D**) Expression levels of each cell stage in the heat-stress group. Values with no common superscript lowercase letters mean significant differences between groups (*p*  <  0.05). (**E**) Effect of heat stress on the expression level of 5hmC in bovine oocytes and embryos. The asterisk (*) denotes significant disparities between groups (*p* < 0.05).

DNA hydroxymethylation (5hmC) staining is shown in Figure 6A,B for both control and heat-stressed oocytes and embryos. Data on 5hmC-expression levels in oocytes and embryos from the heat-stressed and control groups are shown statistically in Figure 6E. The group subjected to heat stress showed a substantial decrease in the fluorescence intensity of 5hmC at all stages of development, indicating a major difference compared to the control (*p* < 0.05).

### 2.4. Effect of Heat-Stress Treatment on ROS Levels in Oocytes

**Figure 7:**
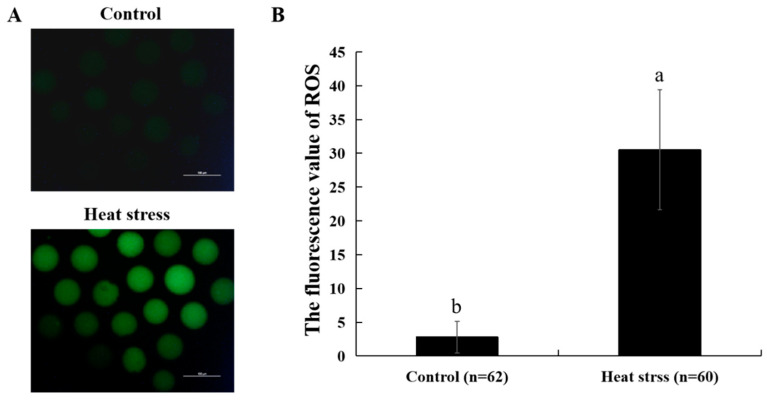
Effect of heat stress on ROS level in bovine oocytes. (**A**) Representative image staining of ROS staining in bovine oocytes. Bar = 100 μm. (**B**) Effect of heat stress on ROS level in bovine oocytes. Values with no common superscript lowercase letters mean significant differences between groups (*p* < 0.05).

Figure 7A shows the representative images of ROS staining in the control and heat-stress groups. Based on the data shown in Figure 7B, the stressed group exhibited a substantially higher production of ROS in comparison to the control (*p* < 0.05).

### 2.5. Effect of Heat-Stress Treatment on Mitochondrial Function in Bovine Oocytes

#### 2.5.1. Effect of Heat-Stress Treatment on the ΔΨm in Bovine Oocytes

**Figure 8:**
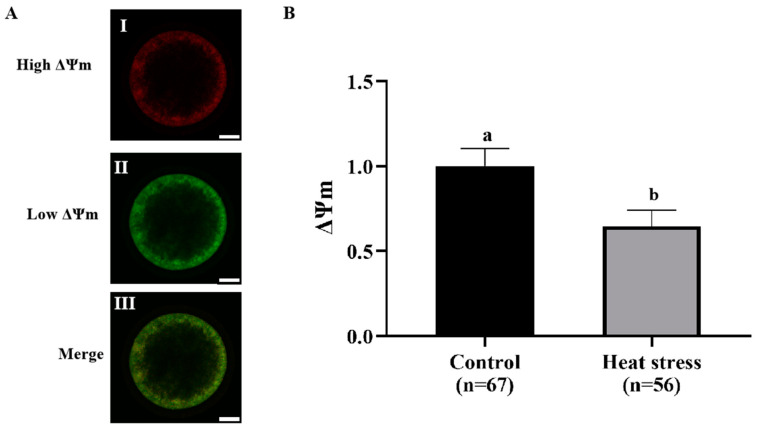
Effect of heat stress treatment on the ΔΨm in bovine oocytes. (**A**) Representative image staining of JC-1 staining in bovine oocytes. I. Red: JC-1 aggregated form (higher ΔΨm); II. Green: JC-1 monomeric form (low ΔΨm); III. Merging of images with green and red fluorescence. Bar = 25 μm. (**B**) Effect of heat-stress treatment on the ratio of red-to-green fluorescence intensity of ΔΨm in bovine oocytes. Values with no common superscript lowercase letters mean significant differences between groups (*p* <  0.05).

Figure 8A shows the representative image of JC-1 staining. As shown in Figure 8B, the ΔΨm level in the heat-stress group was significantly lower than that in the control group (*p* < 0.05).

#### 2.5.2. Effect of Heat-Stress Treatment on the Mitochondrial Distribution in Bovine Oocytes

**Figure 9:**
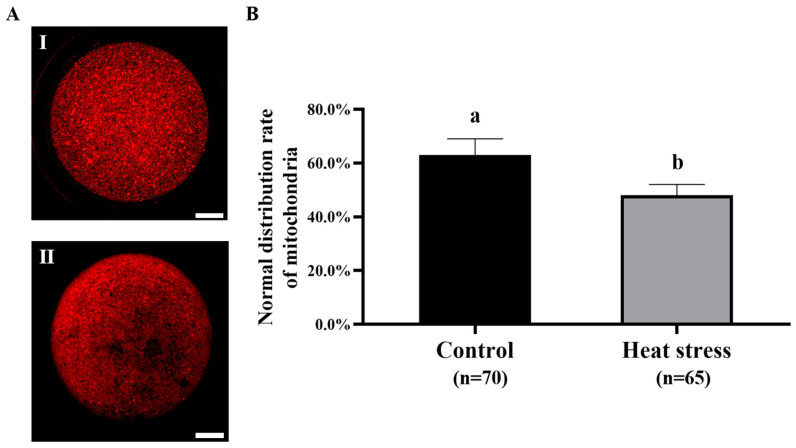
Effect of heat-stress treatment on the mitochondrial distribution in bovine oocytes. (**A**) Mitochondrial distribution. Bar = 50 μm. (I) Normal distribution: mitochondria are distributed throughout the cytoplasm. (II) Abnormal distribution of mitochondria: no mitochondrial signals were observed in some areas of the cytoplasm. (**B**) Effect of heat-stress treatment on the mitochondrial distribution in bovine oocytes. Values with no common superscript lowercase letters mean significant differences between groups (*p* <  0.05).

Figure 9A is a representative image of mitochondrial distribution staining. As shown in Figure 9B, the normal mitochondrial distribution was significantly higher in the control group (62.2%) than in the heat-stress group (43.8%, *p* < 0.05).

#### 2.5.3. Effect of Heat-Stress Treatment on the ATP Level in Bovine Oocytes

**Figure 10:**
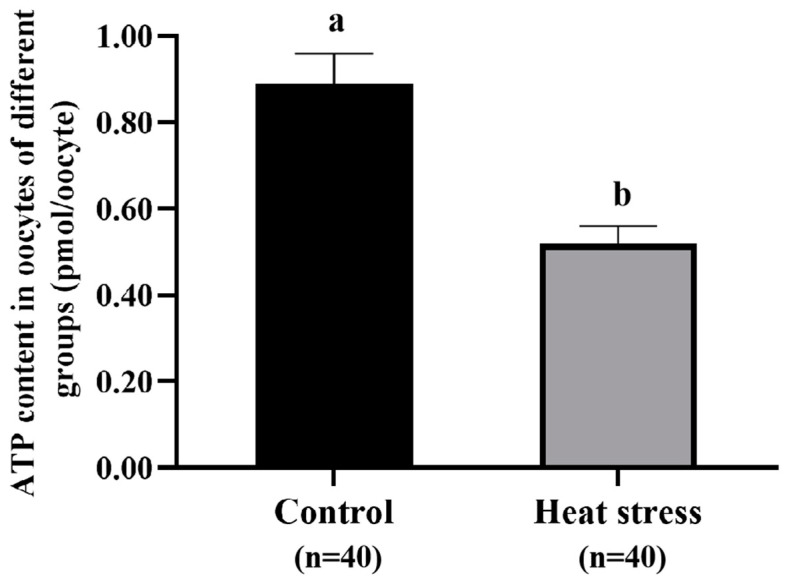
Effect of heat-stress treatment on the ATP content in bovine oocytes. Values with no common superscript lowercase letters mean significant differences between groups (*p* <  0.05).

As shown in Figure 10, the ATP level in oocytes of the control group (0.89 ± 0.07 pmol) was significantly higher than that of the heat-stress group (0.52 ± 0.04 pmol, *p* <  0.05).

#### 2.5.4. Effect of Heat-Stress Treatment on Mitochondrial DNA Copy Number in Bovine Oocytes

**Figure 11:**
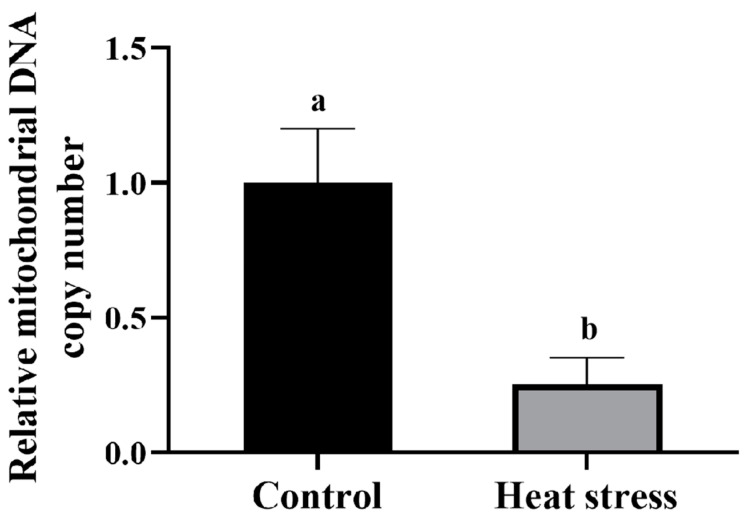
Effect of heat stress on mitochondrial DNA copy number in bovine oocytes. Values with no common superscript lowercase letters mean significant differences between groups (*p* <  0.05).

As shown in Figure 11, the mitochondrial DNA copy number in oocytes of the control group was significantly higher than that of the heat-stress group (*p* <  0.05).

#### 2.5.5. Effect of Heat-Stress Treatment on Mitophagy in Bovine Oocytes

**Figure 12:**
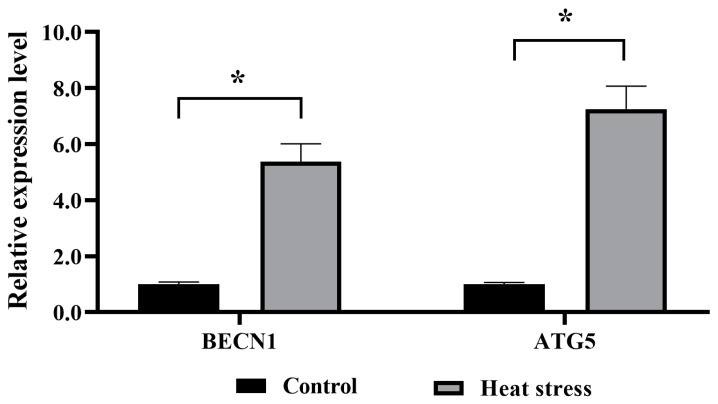
Effects of heat stress on mRNA-expression levels of mitophagy-related genes in bovine oocytes. The asterisk (*) denotes significant disparities between groups (*p* < 0.05).

The changes in two autophagy-related genes (BECN1 and ATG5) in the control and heat-stress groups were observed by quantitative real-time polymerase chain reaction (qRT-PCR) analysis. As shown in Figure 12, the results showed that the expression levels of autophagy-related genes BECN1 and ATG5 in oocytes from the heat-stress group were decreased. This indicated that heat stress induced mitophagy in oocytes.

### 2.6. Effect of Heat-Stress Treatment on Apoptosis in Bovine Oocytes

**Figure 13:**
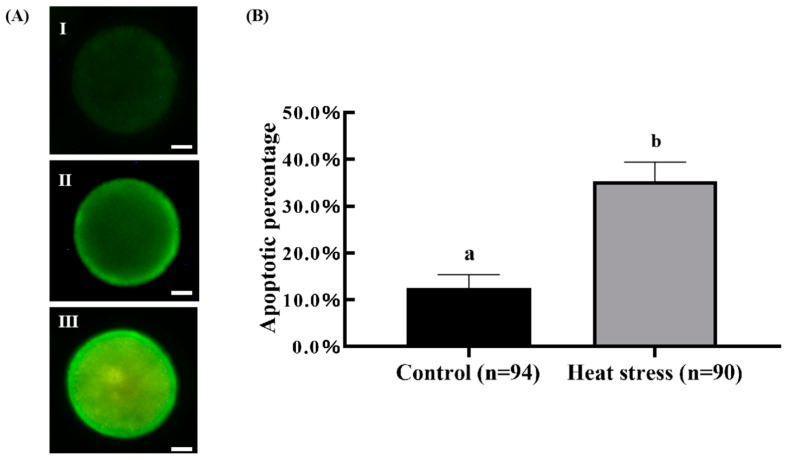
Effect of heat-stress treatment on the membrane integrity of oocytes. (**A**) Representative images of Annexin V staining, scale bar = 25 μm. (Ⅰ) The image of Annexin V-negative oocyte. (Ⅱ) The image of Annexin V-positive oocyte. (Ⅲ) The image of necrotic oocyte. (**B**) Effect of heat-stress treatment on the PS externalization of oocytes. Values with no common superscript lowercase letters mean significant differences between groups (*p* <  0.05).

Figure 13A shows the representative pictures of Annexin V staining. As shown in Figure 13B, the rate of oocytes with PS externalization events in the heat-stress treatment group (35.30 ± 4.12%) was significantly higher than that in the control group (12.53 ± 2.86%, *p* < 0.05).

### 2.7. Effect of Heat-Stress Treatment on TZPs in Bovine Oocytes

**Figure 14:**
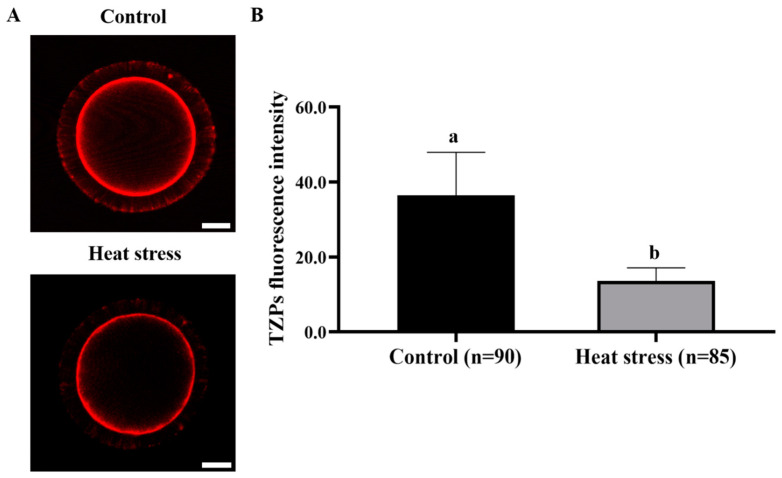
Effect of heat stress on the fluorescence intensity of TZPs in bovine oocytes. (**A**) Representative images showing the staining of TZPs in oocytes. Bar = 50 μm. (**B**) Impact of heat stress on TZPs in oocytes. Values with different superscripts indicate significant differences between groups (*p* < 0.05).

TZP staining in both groups is shown in illustrative images in Figure 14A. As shown in Figure 14B, the TZP level in the heat-stress group was significantly lower than that in the control group (*p* < 0.05).

### 2.8. Effects of Heat Stress on Gene Expression in Bovine Oocytes

**Figure 15:**
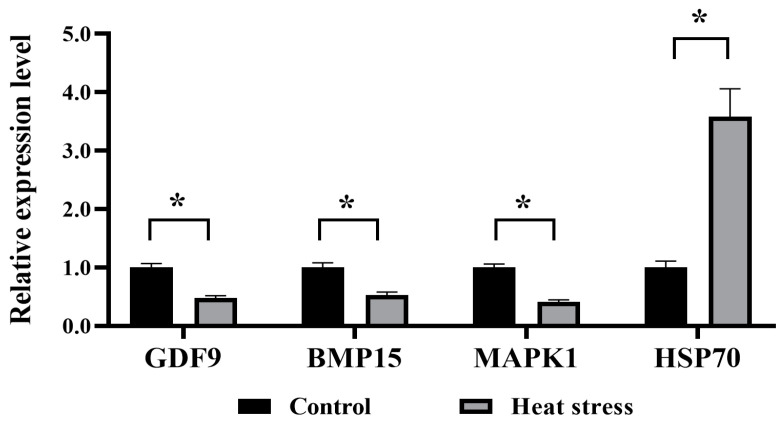
Effects of heat stress on mRNA expression levels of genes related to developmental ability of bovine oocytes. The asterisk (*) denotes significant disparities between groups (*p* < 0.05).

**Figure 16:**
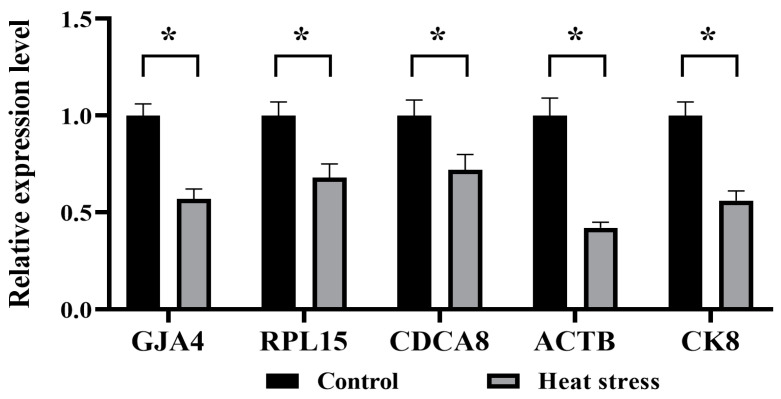
Effects of heat stress on mRNA expression levels of genes related to cytoskeleton of bovine oocytes. The asterisk (*) denotes significant disparities between groups (*p* < 0.05).

**Figure 17:**
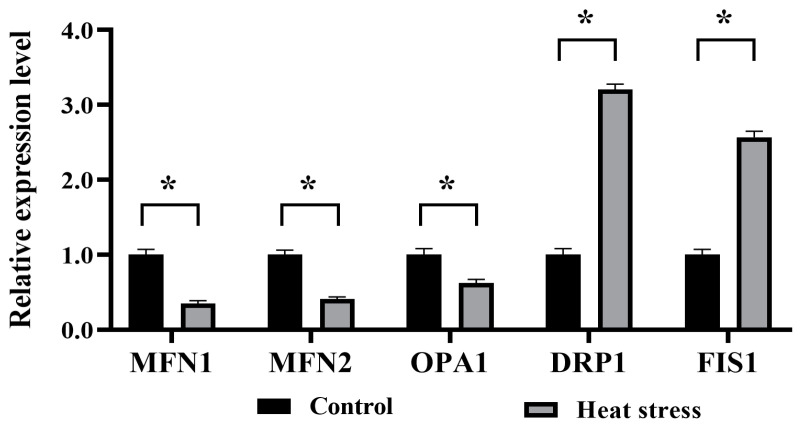
Effects of heat stress on mRNA expression levels of genes related to mitochondrial function of bovine oocytes. The asterisk (*) denotes significant disparities between groups (*p* < 0.05).

The data presented in Figure 15 indicate a pronounced drop in the mRNA expression of *GDF9*, *BMP15*, and *MAPK1* in the heat-stress group in contrast to the control (*p* < 0.05), whereas the heat-stress group had a significantly higher level of *HSP70* mRNA expression than the control (*p* < 0.05). Referring to Figure 16, in comparison to the control, the heat-stressed group had significantly lower levels of mRNA expression for the cytoskeleton-related genes *GJA4*, *RPL15*, *CDCA8*, *ACTB*, and *CK8* (*p* < 0.05). As shown in Figure 17, the mRNA expression of mitochondrial function-related genes *MFN1*, *MFN2*, and *OPA1* in the heat-stress group was significantly lower than that in the control group (*p* < 0.05). Conversely, the group challenged by heat stress demonstrated substantially higher mRNA expression levels of *DRP1* and *FIS1*, in contrast to the control (*p* < 0.05).

### 2.9. Effect of Heat Stress on the Expression of Genes Related to Epigenetic Modifications in Bovine

#### 2.9.1. Effect of Heat Stress on the Expression of Genes Related to Epigenetic Modifications in Bovine Oocytes

**Figure 18:**
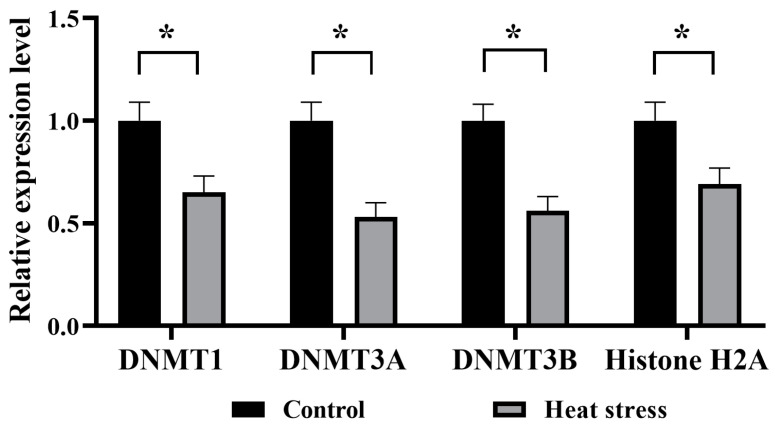
Effects of heat stress on mRNA expression levels of genes related to epigenetic modification of bovine oocytes. The asterisk (*) denotes significant disparities between groups (*p* < 0.05).

As shown in Figure 18, the mRNA expression of *DNMT1*, *DNMT3A*, *DNMT3B* and *Histone H2A*, genes related to epigenetic modifications in oocytes from the heat-stressed group, was significantly lower than that of the control group (*p* < 0.05).

#### 2.9.2. Effect of Heat Stress on the Expression of Genes Related to Epigenetic Modifications in Bovine Blastocysts

**Figure 19:**
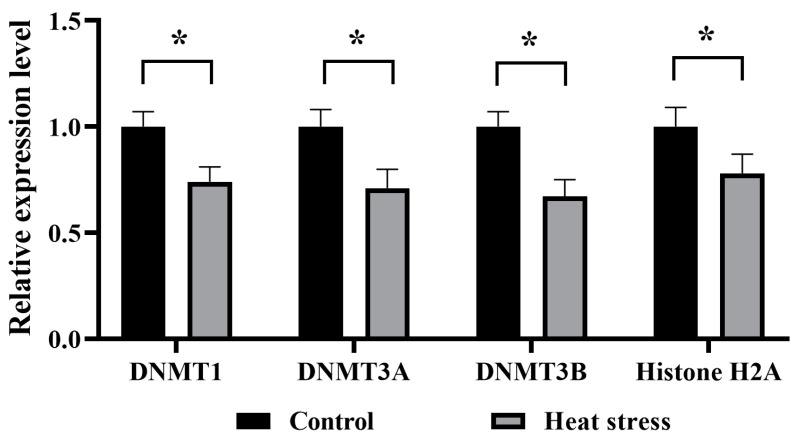
Effects of heat stress on mRNA expression levels of genes related to epigenetic modification of bovine blastocysts. The asterisk (*) denotes significant disparities between groups (*p* < 0.05).

As shown in Figure 19, the mRNA expression of genes *DNMT1*, *DNMT3A*, *DNMT3B* and *histone H2A* associated with epigenetic modifications was significantly lower (*p* < 0.05) in the heat stress group than in the control group.

## 3. Discussion

Summer heat stress disrupts homeostatic reproduction in cattle by affecting fertility and reproductive function [19]. Extensive in vitro studies indicate that 12 h of heat stress (41 °C) during early maturation dramatically reduces COC viability and blastocyst production [28]. It may cause a decrease in the cleavage and blastocyst rates, ranging from 30% to 65% [18]. Similarly, our study demonstrates that heat stress reduces oocyte developmental ability, cleavage rate, and blastocyst rate, as illustrated in Table 1. The vulnerability to heat stress usually persists from the oocyte stage until the cleavage stage, leading to a subsequent decline in embryo developmental potential [9]. Heat stress impairs oocyte development through numerous cellular- and molecular-level aberrations [29]. Consequently, a significant proportion of oocytes that are subjected to heat stress experience a halt in development at the metaphase stage [17]. Thus, our results completely align with the previously reported finding. In addition, it has been shown that the addition of one-carbon metabolism (OCM) enhancers during oocyte maturation significantly increased oocyte mitochondrial mass, DNMT3A protein expression, and blastocyst rate, possibly due to improved epigenetic programming [30]. OCM consists of three related pathways: the folate and methionine cycles, and the trans-sulphuration pathway [30].

Several genes encode histones, and the linker H1 exhibits more variation than the core histone [31]. Mammals exhibit a total of 11 distinct variations of histone H1 [32]. Histone H1 is crucial for preserving the structure and stability of chromatin, and it regulates gene expression by either activating or inhibiting transcription [33]. Tanaka reported that the presence of mouse oocyte-specific adaptor histone H1 in GV-stage oocytes persisted in MII-stage oocytes and 2-cell embryos, but was notably reduced in 4–8-cell embryos [34]. Funaya observed a gradual decrease in the expression of histone H1F0 from the unicellular stage to the metaphase stage and a slight increase at the blastula stage [35]. Fu demonstrated that the reduction in histone H1 levels in the chromatin of mouse oocytes occurs throughout embryonic development [31]. Similarly, as shown in Figure 1, our study showed that the expression level of H1F0 in oocytes and embryonic chromatin and cytoplasm gradually decreased from the oocyte to the morula stage, but increased at the blastula stage, and heat stress significantly affected the expression of H1F0. Epigenetic mechanisms contribute to cellular growth, and the process of epigenetic reprogramming is tremendously influenced by heat stress [36]. As cells develop in the aforementioned conditions, there are changes that occur in the expression of histone H1 [27].

Histone H2A is one of the core histones that make up nucleosomes, and has various variants [37]. It plays a vital role in the regulation of cell pluripotency, differentiation [38], nuclear function and embryonic genome remodeling [39]. Histone H2A variants are involved in the synthesis of unique chromatin structures that facilitate reprogramming events [40]. Bettegowda demonstrated that the expression of total H2A mRNA was highest at the prokaryotic stage of early development, then declined rapidly at the 8-cell stage and remained low from the 16-cell stage to the blastocyst stage [41]. Vigneault showed that the mRNA expression level of histone H2A.1 gradually decreased from the MII-oocyte stage to the morula stage, and it was slightly elevated at the blastocyst stage [42]. It has also been observed that during oogenesis, histone H2A variations do not all congregate in the chromatin, but exist in the cytoplasm [40]. Takahashi reported that the number of H2AX-phosphorylated lesions increased linearly after heat treatment in a heating time-dependent manner [43]. On the other hand, Rogakou found that the ratio of histone variant H2A.1 to H2A.2 in human senescence cells decreased linearly [44]. Our study revealed that the expression level of histone H2A gradually decreased from the MII-cell stage to the morula stage, and slightly increased in the blastula stage, as illustrated in Figure 2. Moreover, the heat-stress treatment group exhibited lower expression levels of H2A compared to the control group.

Histone H2B plays an important role in cell division [45], and the N-terminal of histone H2B is essential for maintaining the cohesion of chromosomes [46]. Histone H2B is also involved in the gene regulatory mechanism [47]. Mouse histone H2B synthesis begins at late 1-cell stage and reaches its maximum rate in early 2-cell embryos [48]. According to Rozinek, in porcine 4-cell embryos, histone H2B is present in heterochromatin and is more strongly expressed in the nuclear periphery [49]. Kafer observed a significant decrease in histone H2B expression from the fertilized oocyte to the 2-cell stage and a gradual increase in H2B expression during development to the blastocyst stage [50]. In the in vitro environment, H2A-H2B proteins are denatured in response to elevated temperatures and cellular functions induce structural changes in H2A-H2B to mitigate heat-induced DNA damage [51]. Similarly, our results demonstrate that histone H2B remains throughout embryonic development and that heat stress decreases its expression.

Histone H4 plays a structural role in the nucleosome, is the most conserved component of the histone octamer, and packages DNA into chromatin during the S phase [52]. The n-terminal tail of core histones (especially H4) undergoes various modifications, and these modifications play a key role in the transcription process [53]. In mammalian oocytes, deacetylation of histone H4 in chromatin during meiotic maturation is associated with chromosome formation [54]. Histone H4 is synthesized during the early stages of development in single-cell embryos [48], and early embryos enrich histone H2A and H4 methylation [38]. Research has shown that histone H4 is found in heterochromatin and has higher expression on the periphery of the nucleus in 4-cell pig embryos [49]. Our findings clearly suggest that histone H4 is present throughout embryonic development, and that heat stress affects its expression and reduces oocyte developmental capacity. Alterations in histone H4 not only result in genetic instability, but also in escalated programmed cell death and aberrant progression of the cell cycle during the first stages of cellular development [55].

Environmental stress has a significant impact on DNA methylation [23], and immunostaining can reveal the dynamic changes in 5mC methylation that occur throughout embryonic development [56]. DNA methylation is a primary epigenetic modification in the genome [24], which plays a critical part in maintaining genomic integrity and supporting cell development [23]. DNA methylation is also an essential aspect that regulates gene expression via several mechanisms, including genomic imprinting, X-chromosome inactivation, and genetic transcriptional silencing of specific genes [57]. Dobbs demonstrated that the immunoreactivity of 5mC decreased from the peak of the 2-cell stage to the lowest point of the 6–8 cell stage, and increased between the 8-cell stage and the blastocyst stage [58]. In normal bovine embryos, DNA methylation decreased significantly between the 2-cell stage and the 4-cell stage; following the 8-cell stage, there is an increase in DNA methylation, leading to the development of de novo methylation [25]. Hu demonstrated that slow freezing and DMSO freezing reduced the DNA methylation level of bovine oocytes [59]. Evidence has firmly proven that oocyte vitrification may cause stress and have a notable effect on the reprogramming of epigenetic factors during embryo development [22]. Similarly, our results confirmed that oocytes in the heat-stress group had lower DNA methylation levels than those in the control group, as seen in Figure 5. DNA methylation is susceptible to heat stress, and modifications in DNA methylation patterns may affect oocyte developmental competence and gene expression [23].

The epigenome of early mammalian embryos undergoes substantial reprogramming to reach its complete developmental potential [60]. The primary initiating event in reprogramming is the demethylation of 5mC in the fertilized ovum, and 5hmC serves as an intermediary in the active demethylation process of 5mC [61,62]. Early-cleavage-stage 5hmC is a rather persistent alteration, and its presence in mammalian cells is linked to pluripotency [63]. In mammalian cells, the 5hmC is an essential part of the genomic DNA and is crucial for reprogramming DNA methylation throughout the whole genome [64,65]. The study found that 5hmC had higher abundance levels in the perinucleolar area, displaying more intense staining in the cortex and central prokaryotic region [61]. Wossidlo demonstrated that 5hmC accumulates in both prokaryotes during fertilized ovum development [60]. The results of Ruzov’s study using immunostaining showed that 5hmC was detected at all stages of mouse embryo development, decreasing at the 8-cell stage and increasing at the morula and blastula stages [63]. The 5hmC content of low-quality human embryos has been reported to be 1.7 times higher than that of high-quality embryos on the third day of embryonic development, decreasing by 1.4 times on the fourth day [66]. Similarly, our results showed that 5hmC expression levels were present at all stages of bovine embryonic development, with a minimum at the 8-cell stage and a gradual increase thereafter. In addition, we noted that the expression of 5hmC was decreased at all stages in the heat-stimulated group compared to the control group. Heat stress during development can cause abnormalities in the epigenome of oocytes and embryos, leading to potentially heritable epigenetic variation [27,36].

The balance between intracellular antioxidant capacity and ROS affects oocyte developmental competence [67]. When there is an imbalance between ROS production and antioxidant capacity, ROS can cause cellular damage [68]. In addition, it leads to the oxidation of proteins, lipids, and even DNA [69]. Heat stress during IVM of oocytes leads to a significant elevation in ROS and lipid peroxide production [70]. Heat stress may harm oocytes by altering their redox state by increasing the ROS level [68]. Previous studies have shown conclusively that levels of ROS in oocytes show a considerable rise under heat stress [71]. Consistent with the previously described findings, our investigations confirmed that heat stress significantly elevated ROS levels in oocytes and reduced their developmental capacity. Heat stress triggers oxidative stress, leading to a substantial increase in ROS levels [11]. This ultimately results in many types of cellular damage and deteriorates the quality and viability of the oocyte [9].

Dysfunction in one or more aspects of mitochondrial biology results in reduced oocyte developmental competence, including reduced ΔΨm, altered mitochondrial distribution, reduced ATP levels, and reduced mtDNA numbers [72]. ΔΨm and mitochondrial distribution are critical for oocyte development and viability, and ΔΨm maintains ATP production by oocyte mitochondria [73]. The number of mtDNA copies per oocyte correlates with oocyte developmental competence, and the amount of mtDNA is positively correlated with the total number of oocytes and survival [74]. Summer heat stress causes a decrease in ΔΨm and impairs oocyte developmental competence [75]. Uniform mitochondrial clusters were present in the cytoplasm of better-quality oocytes, while mitochondrial clusters were more restricted in poorer-quality oocytes [75]. The mtDNA copy number of oocytes in summer was significantly lower than the mtDNA copy number in winter in the same species of heifers, which may be related to mitochondrial dysfunction and reduced fertility in summer [74]. In the present study, we found that heat stress significantly decreased oocyte ΔΨm, ATP levels, and mtDNA copy number, and that mitochondria were uniformly distributed in the cytoplasm of control oocytes, whereas they were not uniformly distributed in oocytes of the heat-stressed group. In addition, mitochondrial autophagy regulates mitochondrial mass through selective autophagy detection and repair of damaged mitochondria to maintain cellular activity and function [76]. *BECN1* is the mammalian homologue of yeast autophagy-related gene 6 (*ATG6*), and induction or phosphorylation of *BECN1* is required for initiating autophagosome formation through activation of class III phosphoinositide 3-kinase (PI3K) [77]. *BECN1* interacts and forms a complex with PI3K, and this complex is required to recruit the ATG12-ATG5 conjugate to the pre-autophagosome [78]. Our results showed that heat stress increased the expression levels of mitophagy-related genes *BECN1* and *ATG5*. This suggests that heat stress induces mitochondrial autophagy.

Apoptosis is a programmed cell death that is involved in the homeostasis maintenance of many biological processes [71]. The Annexin-V binding process measured by the V-FITC assay is associated with early apoptosis, namely the flipping of phosphatidylserine from the inside to the outside of the cell [79]. This binding is more frequent in oocytes exposed to heat stress, suggesting that heat stress during IVM can trigger programmed cell death (apoptosis) in bovine oocytes [28]. Lord demonstrated a significant increase in the proportion of Annexin-V-positive cells in mouse oocytes cultured for 8 h (*p* < 0.05), 24 h (*p* < 0.05) and 48 h (*p* < 0.001) [80]. Kalo documented that Annexin-V binding was significantly increased in the oocytes treated with short-term heat stress for 0.5–2.5 h at the early stage of maturation (*p* < 0.01) [79]. Our findings show that heat-stress treatment of oocytes 12 h before maturation resulted in an enormous rise in Annexin-V binding and an increase in the proportion of oocytes that undergo early apoptosis compared with controls.

TZPs, as the only carrier of contact between cumulus cells and oocytes, are the contact-dependent communication between cells [81]. TZPs play important roles in oocytes, including providing substrates and maintaining products of metabolic activity [82]. TZPs are also essential for maintaining adhesion between the two types of cells, which is necessary to maintain the integrity of the cumulus–oocyte complex [83]. Therefore, TZPs are essential for the normal development of oocytes [84]. Most TZPs have actin-rich cytoskeletons; ghost cyclic peptides bind to and stabilize polymerized actin and are commonly used to label oocyte TZPs [85]. Tseng showed that TZP staining intensity of mature oocytes treated with heat stress at 41.5 °C for 4 h decreased [86]. Our results showed that TZP staining intensity of mature oocytes treated with heat stress at 41.0 °C for 12 h was significantly lower than that of the control group. Reduced TZP levels represent the inhibition of actin polymerization by heat stress, and significant changes in the cytoskeleton after heat stress are associated with reduced oocyte developmental capacity in hot seasons [86].

Gene expression analysis provides a new way to better explore the effects of heat stress on oocyte development by detecting target genes [73]. Studies have shown that oocytes are the main source of *GDF9* and *BMP15*, and mRNA levels of both factors increase as oocytes develop [81]. *GDF9* is involved in the regulation of energy metabolism and cholesterol biosynthesis, and has an impact on ovarian follicle development and ovulation rate [82]. *BMP15* is specifically expressed in oocytes and can improve oocyte development and promote early embryonic development in cattle [87]. *MAPK1* plays a vital role in the maturation of bovine oocytes [88]. *HSP70* protein is the central hub of protein balance and plays an important role in ameliorating stress damage [70]. Gendelman and Roth found that the mRNA expression of the *GDF9* gene in oocytes during a cold winter was significantly higher than those observed in summer [89]. Souza-Cacares showed that when COCs were subjected to high temperature during IVM, this would increase *HSP70* mRNA expression [90]. Similarly, this study also showed that the mRNA expression levels of *GDF9*, *BMP15* and *MAPK1* in oocytes under heat stress in vitro were significantly lower than those in the control group, and the mRNA expression levels of *HSP70* were significantly higher than those in the control group. Heat-stress proteins not only regulate apoptotic pathways and influence oocyte developmental capacity, but also serve as biomarkers for cell stress [90].

*GJA4* is essential for oocyte development, and the deletion of the *GJA4* gene encoding the oocyte gap conjunction can lead to follicular dysplasia and inhibit oocyte development and meiosis [82,91]. The *RPL15* gene encodes a ribosomal protein [92], and the *CDCA8* gene facilitates the production of a protein that regulates chromosomal structure and the cytoskeleton during cell division [93,94]. These two proteins maintain the cell cycle and meiosis process of oocytes [95]. Beta-actin (*ACTB*) is a highly conserved protein, and *ACTB* is associated with cell motility, structure, and integrity [96]. *CK8* plays an important role in regulating the assembly and decomposition of cytoskeletal filaments [97]. Macabelli observed a substantial decrease in the levels of *ACTB* and *RPL15* expression in immature oocytes collected with OPU from heifers during the summer compared to winter [98]. Wei found that the expression levels of the *GJA1*, *ACTB*, and *CK8* genes were significantly higher in the control oocytes at stage MII than in the vitrified group [97]. The research findings demonstrated that heat stress led to a reduction in the mRNA expression levels of many genes, such as *GJA4*, *PRL15*, *CDCA8*, *ACTB*, and *CK8*. This decrease was consistent with the results of heat-stress damage to TZPs.

Uninterrupted fission and fusion processes in mitochondria are essential for maintaining optimal mitochondrial activity, integrity and internal homeostasis [99]. Mitochondrial fusion transfers gene products between mitochondria, thereby optimizing mitochondrial function, especially under environmental stresses such as heat stress [100], whereas mitochondrial fusion is mediated by Mitofusin-1 (*MFN1*), Mitofusin-2 (*MFN2*) and optic atrophy 1 (*OPA1*) [101]. The expression of *MFN1*, *MFN2*, and *OPA1* promotes mitochondrial elongation and further protects cells during cell development or nutrient deficiency [102]. Mitochondrial fission is mediated by dynamin-related protein 1 (*DRP1*) and mitochondrial fission protein 1 (*FIS1*), but the high *DRP1* expression leads to mitochondrial fragmentation, apoptosis and cell death [103]. Multiple studies indicate that extreme heat exposure might potentially cause damage to the form and structure of mitochondria [104], as well as upset the equilibrium between mitochondrial fission and fusion, leading to mitochondrial malfunction [105]. Zeng found that heat stress induced significant down-regulation of mRNA expression of *MFN1* and *MFN2* and significant up-regulation of *DRP1* mRNA expression in bovine mammary epithelial cells (BMECs) [106]. Chen showed that dairy cow mammary epithelial cell (DCMEC) genes *MFN1*, *MFN2*, and *OPA1* were down-regulated, while *DRP1* and *FIS1* were up-regulated after heat stress [105]. Based on our findings, the mRNA levels of *MFN1*, *MFN2*, and *OPA1* were noticeably reduced in heat-stressed oocytes compared to the control group. Nevertheless, the expression levels of *DRP1* and *FIS1* mRNA in heat-stressed bovine oocytes exhibited a significant increase when compared to the control group. Heat stress skews the equilibrium between mitochondrial fission and fusion, which causes cellular malfunction, as explained in previous reports [106].

The epigenetically related gene *DNMT1* plays a crucial role in bovine oocyte and embryo development, influencing key developmental stages in the establishment of the epigenome prior to embryo implantation [107]. Pavani observed a significant down-regulation of the expression level of the *DNMT1* gene in embryos produced in warmer months relative to colder months, which correlates with a reduced ability of oocytes and embryos to develop as a result of the summer season [108]. DNA methylation is mediated by the slave DNA methyltransferases *DNMT3A* and *DNMT3B*, with *DNMT3A* acting with equal efficiency on both hemimethylated and non-methylated DNA during early embryonic development [109]. *DNMT3B* is involved in the methylation of slave DNA and is subject to epigenetic regulation of methylation [58]. Stamperna demonstrated that the heat stress-treated group blastocyst *DNMT3A*-expression level was significantly lower than that of the control group [17]. *Histone H2A* is the most stable reference gene during oocyte development and is used to standardize the measurement of mRNA abundance in bovine oocytes and embryos that are at similar developmental stages and at different developmental stages [41]. The fluorescence quantification results of this study showed that heat stress reduced the expression of epigenetically related genes *DNMT1*, *DNMT3A*, *DNMT3B*, and *Histone H2A*, and decreased the developmental capacity of oocytes and embryos.

## 4. Materials and Methods

Unless otherwise specified, all the reagents consumed in the lab work were lab-grade and procured from reputed vendors at the Sigma-Aldrich Chemical Company (St. Louis, MO, USA).

### 4.1. In Vitro Maturation of Oocytes (IVM)

Ovaries were retrieved from mature cows slaughtered at a local slaughterhouse and delivered to the lab in an insulated box within 2 h. Ovaries remained immersed in a transport medium, saline solution (8.9 g/L NaCL) containing penicillin (0.01%) maintained at 33 °C. On arrival, ovaries were pre-washed thrice in the same shipping medium to reduce contamination. Immature cumulus–oocyte complexes (COCs) were collected by aspirating follicles of 2-to-8 mm diameter with an 18 mm gauge needle, providing vacuum pressure of 80 mmHg. The collected COCs, after pooling, were then graded to obtain the desired quality. Only oocytes with homogenous cytoplasms surrounded by equal to or more than three compact cumulus-cell layers were used for in vitro maturation (IVM).

The IVM medium used in the experiment mainly consisted of M199 (Gibco BRL, Grand Island, NY, USA). The medium was supplemented with follicle-stimulating hormone (FSH) (10 μg/mL), luteinizing hormone (LH) (10 μg/mL), estradiol (1.0 μg/mL), insulin-like growth factor (IGF) (40 ng/mL), and fetal bovine serum (FBS) (10%) (Gibco BRL, USA). A set of fifty COCs was co-cultured on four-well Petri plates using 500 μL of IVM media underneath a mineral oil drop. Those COCs were incubated at 38.5 °C with 5% CO_2_ for 24 h. To induce stress, the heat-stressed group initially underwent a higher maturation temperature (41.0 °C) with 5% CO_2_ for the duration of 12 h. Subsequently, they continued to mature at 38.5 °C for the next 12 h.

### 4.2. In Vitro Fertilization Experiments (IVF)

The technique used for IVF of matured oocytes by Brackett [110] was slightly modified in the current study. It used frozen–thawed semen from a proven sire from the Beijing Dairy Center, Beijing, China, which was processed for fertilization. About 7 mL of Brackett and Oliphant medium was added to the sperm, which was then subjected to two cycles of centrifugation at 1800 rpm for 5 min each. The supernatant liquid was discarded, and the remaining sperm pellet was resuspended in fertilization solution to achieve 1 × 10^7^/mL concentration. Later on, 10 μL of IVF medium was then added, together with 90 μL sperm suspension, and the mixtures were co-incubated for 1.5 h to encourage fertilization. In each drop of fertilization solution there were about 25 oocytes, which were incubated for a period of 16–18 h at the temperature of 38.5 °C and under a CO_2_ level of about 5%. The presumed embryos were placed in CR1aa media for 48 h after fertilization. Subsequently, they were incubated in CR1aa media supplemented with 10% FBS for a period of 5 days, and half of the medium was renewed after every 48 h.

### 4.3. Immunofluorescence Analysis

Oocytes and embryos were evaluated by immunofluorescence assay as reported by Funaya [35], with slight modifications. The mature oocytes at the MII stage were collected 24 h after oocyte maturation, and embryos at the 2-cell, 4-cell, and 8-cell stages were gathered 36, 48, and 72 h after fertilization, respectively. Similarly, the embryos at the morula and blastula stages were sampled on day 6 and day 8 of development. The oocytes and embryos were subjected to three washing cycles in a 0.1% PBS-PVA solution, each cycle lasting for 5 min. After that, the oocytes and embryos were subjected to immersion in a 4% paraformaldehyde solution that was kept overnight at 4 °C for fixation. After permeabilization with 0.5% Triton X-100 in 0.1% PBS-PVA for 40 min at room temperature, the cells were closed with 1% BSA in 0.1% PBS-PVA at 4 °C overnight. Oocytes and embryos were incubated overnight at 4 °C with primary antibodies H1F0 (Solarbio, Beijing, China, 1:200), H2A (Bioss, Beijing, China, 1:500), H2B (Solarbio, Beijing, China, 1:700), H4 (Easybio, Beijing, China, 1:500), 5-methylation (Epigentek, Boston, MA, USA, 1:200) and 5-hydroxymethylation (Epigentek, Boston, MA, USA, 1:200) and then washed three times with 0.5% Triton X-100 for 10 min each. Then samples were treated with secondary antibodies, Alexa Fluor-488 (anti-rabbit; Solarbo, Beijing, China), Alexa Fluor-647 (anti-rabbit; Abcam, Cambridge, UK), Alexa Fluor-488 (anti-mouse; Bioss, Beijing, China), Alexa Fluor-680 (anti-rabbit; Abcam, Cambridge, UK), Alexa Fluor-488 (anti-mouse; Bioss, Beijing, China) and Alexa Fluor-594 (anti-mouse; Bioss, Beijing, China). The antibodies were diluted at a ratio of 1:500 at room temperature for 1 h. Then, the nuclei were incubated with 4′, 6-diamidino-2-phenylindole (DAPI; Invitrogen, Carlsbad, CA, USA), and the cells were fixed on a glass slide for examination under the confocal laser microscope (Leica, Wetzlar, Germany).

### 4.4. Oxidative Stress in Oocytes

The protocol of Rahimi [111] was adopted with slight modifications to quantify intracellular ROS production levels in oocytes. The surrounding granulosa cells were stripped off after a maturation period of 22–24 h by immersing the COCs in a solution containing hyaluronase provided in a concentration of at least 1 mg/mL and using repeated gentle pipetting with the help of micropipettes. After this, the oxidative stress in the oocytes was evaluated using mini commercial ROS detection kit (S0033S, Beyotime, Shanghai, China). Then the oocytes were transferred to 10 μmol·L^−1^ DCFH-DA staining solution and left for incubation for 20 min at the temperature of 37 °C in dark conditions. After staining, oocytes were washed three times with 0.1% PVA-PBS solution, and then observed on a laser confocal microscope (Leica, Wetzlar, Germany) and photographed. In addition, the Image J software version 1.8.0 (NIH, Bethesda, MD, USA) was used to measure the fluorescence intensity of the digital images.

### 4.5. ΔΨm Examination of Oocytes

The detection of oocyte ΔΨm was performed by JC-1 staining, which was slightly modified by referring to the method of Gendelman [75]. After maturation for 22–24 h, the oocytes were placed in 1 mg/mL hyaluronase solution to remove granulosa cells. Subsequently, they were washed using a 0.1% PVA-PBS solution. After the washing step, the oocytes were incubated in JC-1 solution (10 μg/mL) and kept at 37 °C for about 20 min. Afterwards, they underwent washing with 0.1% PVA-PBS solution. A confocal laser scanning microscope scanned the stained oocytes and digital images were captured. Furthermore, the relative intensity of fluorescence in the captured digital images was assessed using Image J software version 1.8.0 (NIH, Bethesda, MD, USA).

### 4.6. Mitochondrial-Distribution Examination of Oocytes

The mitochondrial distribution of the oocytes was assayed by staining with MitoTracker Red CMXRos (M7512, Invitrogen, Carlsbad, CA, USA), with a slight modification to the method of Lee [112]. After 22–24 h of maturation, the oocytes were placed in 1 mg/mL hyaluronidase solution to remove granulosa cells. Subsequently, the oocytes were washed with 0.1% PVA-PBS solution. After the washing step, the oocytes were placed in MitoTracker Red solution (100 nM) and incubated at 37 °C for about 30 min. Then, the oocytes were washed with 0.1% PVA-PBS solution. After pressing, the stained oocytes were scanned with a confocal laser scanning microscope (Leica, Wetzlar, Germany) and digital images were captured. The relative intensity of fluorescence in the captured digital images was then assessed using Image J software version 1.8.0 (NIH, Bethesda, MD, USA).

### 4.7. ATP Examination of Oocytes

Oocyte ATP content was determined by the method described by Zhao [113] using an ATP-dependent luciferin–luciferase bioluminescence assay (ATP Bioluminescence Kit HS II, Roche Diagnostics GmbH, Mannheim, Germany). In brief, 10 oocytes were homogenized and lysed with 20 μL of cell lysis reagent. A total of 100 μL of ATP assay solution was added to each well of a 96-well plate, and then the sample and the standard solution (20 μL) were added to the wells. Luminescence was measured immediately for 10 s using a luminometer (InfiniteM200, Tecan Group Ltd., Männedorf, Switzerland). Finally, a standard curve was generated from the relative light intensity of serial dilutions, and the amount of ATP in the sample was calculated from the standard curve.

### 4.8. Detection of Mitochondrial DNA Copy Number in Oocytes

Total DNA (mitochondrial and nuclear) was extracted using a TIANamp genomic DNA kit (TIANGEN, Beijing, China), according to the manufacturer′s instructions. Mitochondrial DNA copy number was determined by real-time polymerase chain reaction using an ABI 7500 SDS instrument (Applied Biosystems, Foster City, CA, USA). The PCR reaction mixture (10 μL) was prepared with 2 μL cDNA, 0.5 μL primer (10 μM), and 2 × SYBR Green Mix (Q121, Novozymes, Beijing, China), and finally ddH_2_O was added to 10 μL. A ratiometric assay of the levels of a single-copy mitochondrial gene, cytochrome c oxidase (*COX1*; primer sequences: forward, 5′-CCTCAATTTTAGGAGCCATCA-3′; reverse, 5′-CTGCTAATACAGGGAGCGAGA-3′), against a single-copy nuclear gene, nth endonuclease III-like 1 (*NTHL1*; primer sequences: forward, 5′-GAAAAGCTACAGCCCCGTGAA-3′; reverse, 5′-GGATGGTGCCTGGAGATGC-3′) was used to estimate the average copy number of mtDNA/nuclear DNA (nDNA). The real-time polymerase chain reaction parameters were as follows: initial denaturation at 95 °C for 5 min, followed by 44 cycles at 95 °C for 10 s and 60 °C for 30 s. The melting curves were analyzed to validate the PCR. Melting curves were analyzed to verify the specificity of the PCR products. Ct values for *NTHI1* were subtracted from those for *COX1* to give ΔCt. Average mtDNA copy number per nuclear genome was calculated as 2 × 2^(ΔCt)^ [114].

### 4.9. Oocyte Apoptosis Assessment

In parallel with the previous analysis, after maturation for 22–24 h, the COCs were placed in 1 mg/mL hyaluronidase solution and repeatedly blown to remove the granular cells. The oocytes were stained with a commercial Annexin V-FITC apoptosis detection kit (C1062M, Biyuntian, Beijing, China). According to recommendations, 100 μL of stain drops was prepared by mixing Annexin V-FITC, propyl iodide stain solution and Annexin V-FITC binding solution. Subsequently, the oocytes were washed thrice with 0.1% PVA-PBS solution and transferred into the stained drop for incubation at room temperature for a duration of 20 min. Following that, the oocytes were again washed thrice with 0.1% PVA-PBS washing solution. The oocytes were later observed under an inverted fluorescence microscope (Leica, Wetzlar, Germany). The photographs were also analyzed by the Image J software version 1.8.0 (NIH, Bethesda, MD, USA).

### 4.10. Detection of Transzonal Projections in Oocytes

The oocyte TZP immunofluorescence staining was conducted by following the previously published protocol by Macaulay [85]. The oocytes were washed thrice in 0.1% PBS-PVA solution. After washing, they were fixed with paraformaldehyde for 30 min. Then, these oocytes were made permeable by using 0.1% TritonX-100 solution for 15 min. Finally, sealing of the oocytes was carried out by incubating them in a solution containing 1% BSA for one hour. Finally, the oocytes were incubated with a concentration of 5 μg/mL rhodamine phalloidin dye (Sigma, Louis, MO, USA) for the next 40 min. The stained oocytes were photographed digitally using a confocal laser scanning microscope (Leica, Wetzlar, Germany). The fluorescence intensity from the TZP-staining digital images was analyzed using the Image J software version 1.8.0 (NIH, Bethesda, MD, USA).

### 4.11. Quantitative Real-Time Polymerase Chain Reaction (q-PCR) of Candidate Genes

The qRT-PCR technique was used to measure the levels of candidate gene expressions and mitophagy. Briefly, the matured COCs were denuded of oocytes by using 1 mg/mL hyaluronidase. The commercial TRIzol kit (Invitrogen, Carlsbad, CA, USA) was used to isolate the total RNA content in the oocytes, under instructions from the manufacturer. Whole RNA content was further reverse-transcribed to produce the cDNA copies using random generating. Furthermore, qRT-PCR was run using purified mRNA (ABI 7500 SDS; Applied Biosystems, Foster City, CA, USA). The reaction took place at 95 °C for 2 min and there were 40 cycles at 95 °C 10 s and 60 °C 30 s. Folding changes in gene expression were analyzed by the 2^−ΔΔCt^ method. The GAPDH gene was considered as an internal standard reference gene, and results were presented with a ratio relative to GAPDH levels. Furthermore, the PCR primers used in the assessment are listed below, in Table 2.

### 4.12. Experimental Design

The effect of summer heat stress on the developmental competence of bovine oocytes was investigated. Expression levels of epigenetically modified histones H1, H2A, H2B, H4, DNA methylation and DNA hydroxymethylation were determined in bovine oocytes and embryos. Subsequently, oocyte ROS, ΔΨm, mitochondrial distribution, ATP levels, mitochondrial DNA copy number, mitophagy, apoptosis, and TZP levels were determined. In addition, expression levels of genes related to developmental competence, the cytoskeleton, and mitochondrial function were analyzed to assess oocyte quality.

### 4.13. Statistical Analysis

All experiments were repeated at least three times and the results were expressed as mean ± standard error. The arcsine transformation of the percentages was performed before analysis. A normal distribution test was performed before data analysis. All data were analyzed by one-way analysis of variance (ANOVA) by Duncan’s test, using SAS software version 9.2.0 (SAS Institute, Carrey, NC, USA). Values with *p* < 0.05 were considered statistically significant.

## 5. Conclusions

Heat stress leads to decreased expression of histone modifications and DNA methylation in oocytes and embryos, which reduces the developmental competence of oocytes. Heat stress also resulted in increased levels of reactive oxygen species, decreased ΔΨm, abnormal mitochondrial distribution, decreased ATP, and decreased mtDNA copy number, and induced mitochondrial autophagy, increased apoptosis, and decreased transzonal projections. In addition, heat stress affected the expression levels of genes related to oocyte developmental competence, the cytoskeleton, mitochondrial function, and epigenetic modifications.

## Figures and Tables

**Table 1:** Effect of heat stress on the development ability of bovine oocytes after IVF.

Groups	No. of Oocytes	No. of Mature Oocytes (%)	No. of Cleavage Embryos (%)	No. of Blastocysts (%)
Control	810	84.07 ± 7.52% (681/810) ^a^	80.32 ± 1.75% (547/681) ^a^	42.05 ± 1.06% (230/547) ^a^
Heat stress	1484	61.99 ± 5.46% (920/1484) ^b^	58.91 ± 2.95% (542/920) ^b^	23.25 ± 1.56% (126/542) ^b^

^a, b^, values with different superscripts indicate significant differences between groups (*p* < 0.05), the same as below.

**Table 2:** Primers used for qRT-PCR of candidate genes in oocytes.

Genes	Primer Sequences (5′-3′)	Size (bp)	GenBank Accession No.
GAPDH	F: AAGGTCGGAGTGAACGGATTC	90	NM_001034034.2
	R: ATTGATGGCGACGATGTCCA		
GDF9	F: GACTCCTCAGTGCCAAGACC	195	NM_174681.2
	R: CAGGTGCACGGCATTTACAC		
BMP15	F: CCTAGGGAAAACCGCACCAT	93	NM_001031752.1
	R: TATGTGCCAGGAGCCTCTGA		
MAPK1	F: CCGTGACCTCAAACCTTCCA	187	NM_175793.2
	R: GATGGACTTGGTGTAGCCCTTG		
HSP70	F: GGGGAGGACTTCGACAACAG	192	NM_203322.3
	R: GAAGTCGATGCCCTCGAACA		
GJA4	F: CTCCGGCCGACTTGCG	186	NM_001083738.1
	R: CCAGGCCCAGGATGAGAATG		
RPL15	F: CGTCAGGATATTCGCCGCTT	136	XM_005226176.3
	R: TACTTGTAGGCGCCCATGTC		
CDCA8	F: AGTGCAAATGCGATCCAAGC	185	NM_001083652.1
	R: TATCCAAGTCCGCTGTTGCT		
ACTB	F: AAGGACCTCTACGCCAACAC	154	NC_037352.1
	R: CACGCCTATCTGCACCGTC		
CK8	F: CAGCAAATGTTTGCGGAATGAATG	71	NM_001256282.2
	R: GAACCAGGCGGAGATCCCTTC		
MFN1	F: AAAGGCTCACTTGGACCACC	144	NM_001206508.1
	R: AAGTGGTTGCCATTTCCTGTTG		
MFN2	F: AGGCTAGGAAGGTGAAGTAACTCAG	181	NM_001190269.1
	R: TGGTACAACTGGAACAGAGGAAA		
OPA1	F: GCCTGACATTGTGTGGGAGA	160	NM_001192961.1
	R: TCCAGGTGAACCTGTGGTG		
FIS1	F: CTGTGGAGGACCTGCTGAAAT	143	NM_001034784.2
	R: CAGAGCAAGGCCTTTACGGA		
DRP1	F: CCAAGTGCATGAGCAGAACC	171	NM_134850.3
	R: AGATTGACCGGCTTCACTGG		
ATG5	F: ACATCTTAGGGTTGGTGGTACA	83	NC_037336.1
	R: AAGGGGTGACCAAAGGTAGC		
BECN1	F: GCTGAAACCAGGAGAGACCC	117	NM_001033627.2
	R: GTGGACATCATCCTGGCTGG		
DNMT1	F: AGTGGGGGACTGTGTTTCTG	218	XM_015471992.2
	R: TGCTGTGGATGTACGAGAGC		
DNMT3A	F: AGCACAACGGAGAAGCCTAA	245	NM_001206502.2
	R: CAGCAGATGGTGCAGTAGGA		
DNMT3B	F: GAGAATAAGACGCGGAGACG	146	NM_181813.2
	R: ACATCCGAAGCCATTTGTTC		
Histone H2A	F: GCGGTCTTGGAGTACCTGAC	204	BF076713.1
	R: AGTCTTCTTCGGGAGCAACA		

## Data Availability

All the data supporting the conclusions in this article have been presented in the manuscript.
